# Supplementary material for: Patterns of Intron Gain and Loss in Fungi
Source: PLoS Biol. 2004 Nov 30;2(12):e422. doi: 10.1371/journal.pbio.0020422 (PMC532390; doi:10.1371/journal.pbio.0020422)
Supplement: Table S1 — Also available at http://genes.mit.edu/NielsenEtAl/. (4.3 MB ZIP). [file pbio.0020422.st001.zip › NielsenEtAl/html/712.html]

AN7678.1.NCU06638.1.MG00760.1.FG10095.1


```
 CLUSTAL W (1.82) Multiple Sequence Alignments - Introns Inserted


Sequence 1: NCU06638.1	1300 aa
Sequence 2: MG00760.1	1478 aa
Sequence 3: FG10095.1	1399 aa
Sequence 4: AN7678.1	1452 aa
Alignment Length: 1592 aa
Number Identitical Residues: 470 aa
Alignment Score (without introns) 22424


MG00760.1 	MHFECTVCLAVARTGAVND2HLLSCCTRVNWVQDLSQHGVRLSKPSIGMAPCVDRSTSRP
NCU06638.1	-------------------~----------------------------------------
FG10095.1 	-------------------~-----------------------------MSREHKAPAGP
AN7678.1  	-------------------~--------------MPEKLSDQHTAGKGQLRQVQSRPSSP
          	                                   ..      ... .        .: .

MG00760.1 	VVRIHGTSK0L~TSTVAATIDGLAPLIFGALEYE0YSTYSSSSSTTIELDVTRLPRLLID
NCU06638.1	---------~-~----------------------~-------------------------
FG10095.1 	RPANFSPTR~L2GKTTNPQPRPPLPSTNPGFASD~DPMLSATPSSNHSLFSS------FS
AN7678.1  	LGNENAALA~D1PTTPGRRRLQKHSSTTFLTDAS~SNNFAGSRLSNLSKRRLSIRDQRVP
          	     ..      .:         .        .     :.:  :. .            

MG00760.1 	RDESQPARNSI~FNSTAFLSFSYGEGFFGSSWLASSRTVPDE0SSLESAS--TDFKLTQF
NCU06638.1	-----------~------------------------------~-----------------
FG10095.1 	RDGEQNQLNDS~TASFDFLPSVSFDDLH--------------~SSIESAS--TDFKLAQF
AN7678.1  	QGPRPQEQPRS2SGAVHHSGNSSVDSTTGSLKNSNSRISLRS~TSMSNLANQNTAENMEF
          	 .            :         ..  .:   :.:     . :: .. :...  .  . 

MG00760.1 	PSPTGQGSILEAHGMA-STIPEKNNIT--QYGSSTRVAMSQTQPQVQTSTRIGTAGSIPR
NCU06638.1	-------------------MGSHSDIS--INATGARVAVQQPAP-----TRTARSGSILR
FG10095.1 	PSSTGEGGSLDAHGADDSSMSSRSQIAPSSNGAANRPAVQSS-------TRPARSSSILR
AN7678.1  	LAPVNFDDFHNSIIAEPSLNHFPLPTTGGTGNDRQSTSFHPTNPWAKNNIDNNTPGPAKG
          	 :. . ..  .:     :        : .        :.  . .  ...     ...   

MG00760.1 	RPSNASTRSFAMPTSASTGSLASGPSIEGAPTAPAAMRARRQSHYPPVSNSN-IAKPPRK
NCU06638.1	RPSITN----GTSRQAGLGTTASNTSGVTTPTAPAAIRTRRPNHYPPVSSTNNTMKAPRR
FG10095.1 	RPSNSIR---------QPNNTTGVSVQTEIATGPGTARSRRQSQYPPVSGTT-VAKPPRK
AN7678.1  	YPSRRKS---------LIGQNISSDSSSRPQASTAAGRNRRQSLIQNQAPDNPTPRAPRK
          	 **               .   .        :...: * ** .     :  .   :.**:

MG00760.1 	SVGPGVLE-SEYGARNTARRRPSLTTSMERNMPSAGRSSIDEGARLIFGDPTRSSTSSRA
NCU06638.1	SAGSDVLG-NSD--AEARRRRPSVASLGERNGTEPVRTSVSVD-----GGPRPMPESTRT
FG10095.1 	SIGPGIIGDGDYGSRPAQKRRPSLYSEGAPTGSARVSMDANPS---WLENTTRNFSTSRA
AN7678.1  	SIGPGFLPTASSARRQSLSSRKASADTTRPDQTSYLRPRSQHHEMNGFNGPR-LPSNARS
          	* *...:   . .   :   * :         .       .        ..     .:*:

MG00760.1 	SKAKSVGPPPRVS-QATLSPDT---SIGSEHFPVGGTMPRSP------RFPGQNSA-KRQ
NCU06638.1	SKARSVQPPPRGSDGATLAHDLSALSLGSGSSPV--TFPRSPKT--AVKAPVPSST-KRI
FG10095.1 	TKAKSVQPPPRSN-QANLGFGN---TLTPEQNRHSTLAPRSPRV--GGRLSTPSSTNKRV
AN7678.1  	AKAKSFQPPSSELGDNFLTTTG----LGDHSRSSSTNAVRTPMKSTAGRVPTPTSSAKRV
          	:**:*. **.   .   *        :       .    *:*  ::. : .  .*: ** 

MG00760.1 	SVIPGV----VATGLGARTISPTDSRRMKRMSLKAPPT------STPQVPTVQATVADAR
NCU06638.1	SVMPGVPHTSHATGLGARTVSPTDTKLMKRMSHNQPSQ------APAPVPTAPMPLATSR
FG10095.1 	SMMPGAHHSSHATGLGARTISPTDTRRMKRMSTMPQSVSLGQVSTPPPPPPVSMNNQVER
AN7678.1  	SVMP-----LHATGLGARTISPTDARRLKRLSTAPQAHPLP---NTPPSQTEPLPHRP-R
          	*::*       ********:****:: :**:*    . .      ..   .        *

MG00760.1 	ASSRSPSMLPRKTS-TPSSSRTTPDP-NRKSYSSGLSINSVNSFNTVRTSTGSMQ-RPAQ
NCU06638.1	PSSRSPSMLPRKIP-TPASSRTTPDVSIRKSYSSGLSAASANSYNTVRSSTGSMQPRVPQ
FG10095.1 	AESKSPSMIPRRTSGTPSSSRTTPDM-HRKSYNSVLSATSCLSINSARTSTGSLNRISAS
AN7678.1  	SSTYSPSNIPRKSA-TPSSNRTTPDP-NRKSYSSGLSLSSNTSYNSARNSGGSLQTRLSQ
          	..: *** :**: . **:*.*****   ****.* **  *  * *:.*.* **::   ..

MG00760.1 	N-GTSRLPAPKALSVHNQANQEDEEDVPPVPAIPKAYESPKDSPAECSFLDKRKSTL-VF
NCU06638.1	S-GNTRLPAPKGVPPQNTANGEEEEEVPPVPAIPKLY----DSPGELSFLDKRKSNR-AF
FG10095.1 	S-STSRLPAPKHTTVHNPIPTDDDEYVPPVPAIPKAYESPKDSPADTYFMEKKKTALSNM
AN7678.1  	NFSSSRLPTPKPRVEHAMNN---AEEVPPVPAIPKAYESPKGESDQPQFSAPRKSSL---
          	. ..:***:**    :        * ********* *.:..... :  *   :*:     

MG00760.1 	DSSSIHSTSTASLSTYPSLEQP-GKVSRKSSIRKVSHQNITLDLDKKGNVSQSKKNLQPL
NCU06638.1	DTSSINSTSTGSVSGAQLTDPPPAKLHRKPSQRKSVHTST---VDKKSSSANSKKNLQPL
FG10095.1 	DAMGANSNPTGVISMPVFPEPT--KLHRKPSGQRSYVPMVAAEPDNKATPPN-KRHLQPL
AN7678.1  	-PLEIELLRSTHISESKSTSGQ------RNSEVLDERGIKTPEAKPHTSVAFGKKNMK--
          	 .   .   :  :*     .        : *         : . . : . . .*::::  

MG00760.1 	RLPPLTLGPLSTPTAAKIAALQEQGSAQSLSATPPPNRRNSKTPTTPMTASKSSFFSRHR
NCU06638.1	RLPPLALGPLSTTTAAKIAALQEQ-NASDRSLSPPPGRQIAKTPSTPMTASRSAYFTTTR
FG10095.1 	RLPPMNLGPLSTPTTNKIAALQDH-NSVDRHETPPPPRHLPKTPTTPMTASKSSFFAKTR
AN7678.1  	-LPPLNLLPLGTPMTSKIEALVDK--DDHTDPSTPSAQHVSKTPSTPLTASKANYFSFRD
          	 ***: * **.*. : ** ** ::        :.*. :: .***:**:***:: :*:   

MG00760.1 	HADKAEGANLRSSSSIYNIRTESPGQAIDTNSTDSIKAINVRQPAQKPSVSPYLSSSLPK
NCU06638.1	NEDKPTVQHLRSTSTIN----RGKGGSMDSYSSNESMGGVVSNVMPQSGMSPYILSSAVK
FG10095.1 	RGDTPELPSLRSSSSVH----HAHFTPTPPDVSSTESSLAYKELDHKQSMSPFLSSSLPK
AN7678.1  	DEDADPLPQARSSTSHFALSTSSSITLRAASSSSALASHDGHTPGGARTVSPYVSYALPK
          	  *       **:::     : .      .  :.   .           :**::  :  *

MG00760.1 	GGLEGSGAFTQQRRTKTGNGFSNGADVSVGTTITQQRPSGPRE--PAASKPRAPKSPGPL
NCU06638.1	GGNQES---TYSNRPKTGD-----PDASVPT----QKPSGPRAPKPAAAKSSTAKSPPPP
FG10095.1 	GGADHG----FLKRSKTGADFTT-SDASIFQDQPHHKPAGPRA--PKDAESTSSKSPPPE
AN7678.1  	SNSEYN----HLRQKPSAD-----YSSRISQS---YKLTGPRP----QTRSSGLSNSETV
          	.. : .      .:  :.       .  :       : :***      :..   ... . 

MG00760.1 	SSPDEPPTPSSMS-SLRRKLSLSWKRSASKASILAGGDKPFDQHAT---ESMPPPRLPAS
NCU06638.1	TDPEEPPTPSSIGTSIRRKLSLSWKRSASKNGNNRPEKVDSSSVRH---DSMPPPRIPIS
FG10095.1 	PTPEEPQTPSSMS-SIRRKLSLSWKRGNSKNNTTAAGDVPDKTHAKQPEEPMPPPRIPIS
AN7678.1  	SIPSDPDSNGLPTTTLRNKITLSRKRSTSKTQQALNAN--TDPKKS---DLMPPPKLPAS
          	. *.:* : .   :::*.*::** **. **       .   .       : ****::* *

MG00760.1 	STMNQLAK--KPPSPSSSSKANGNSSYLESRRRKSSASSLNAIVAQDRVRSDIWGSKNE-
NCU06638.1	ATLGSMPSTTKAPSPTPSSKSNGTGTYLESRRRKSSASSLNAIVAGDRHRGEAAAGKKE-
FG10095.1 	ATLNNLSS-VKQASPSPNMK-QGPSGYLDTRRRKSSGGSLNGLITHDKTKSDSWTAKKP-
AN7678.1  	ATWNNLST-GKDSSPT------LKPSYLKSRRQSSLTNVANPALRKPSFSSEQSLSLSPT
          	:* ..:..  * .**:          **.:**:.*  .  *  :      .:   . . :

MG00760.1 	-SGQSNTSDRPPLSHSSSVMQKILKPRAAPNTNRR-QDVWTADLDKDDLIAEEEMRKFGS
NCU06638.1	-SSLDTVAERS--IHTYSSAQKILKPKSSGATLSQPRDVWTADLDKDDMVAEEEMKKLGS
FG10095.1 	-VPDPSTMPVG---RNTSVMQKILRPRNS-SGLAKGPDSYSADLDKDDQAAEEEMAKLGS
AN7678.1  	VSRESHESDNSQHRSASSILSPVHKIISSAKSNMAATSQTENRIETEIAIADEEMRRLGT
          	                 *  . : :   :        .     ::.:   *:*** ::*:

MG00760.1 	RRKETELAAKTLDALRKRASPKERVSPQDAIRIAMLNIYERGEIVDYNDIYFCGTQNAAK
NCU06638.1	RRKETEMAARTLDALRKRATAKERVGPQEALRIAVLNIYERGEIVDYKDVYFCGTQNAAK
FG10095.1 	RRKETDVAARTLDALKKRATPKERVGAHEAIRIAMLNIYERGEIVDYTDVFFCGTQNAHK
AN7678.1  	KRKDFEKAARELDELRNRAGPKDHVNPAQALRVAHLNIFERGEIIDFKDIYFCGTQDAKK
          	:**: : **: ** *::** .*::*.. :*:*:* ***:*****:*:.*::*****:* *

MG00760.1 	VVGDLKSSIPNFGYDDERGDYSIVPGDHLAYRYEIIDVLGKGSFGQVVRCIDHKTGVLVA
NCU06638.1	VVGNVQSDLPNFGYDDERGDYSIIIGDHLAYRYEIIDVLGKGSFGQVVRCIDHKTGVLVA
FG10095.1 	VVGDLQSDAPNFGYDDERGDYTIVPGDHLAYRYEIIDVLGKGSFGQVVRCIDHKTGGLVA
AN7678.1  	HVGDLNQQSTNFGYDDDRGDYNIVLGDHLAYRYEVVDVLGKGSFGQVVRCIDHKTGNLVA
          	 **:::.. .******:****.*: *********::******************** ***

MG00760.1 	VKIIRNKKRFHQQALVEVNILQKLREW0DPKNKHSMVNFTHSFYFRGHLCISTELLDMNL
NCU06638.1	VKIIRNKKRFHQQALVEVNILQKLREW0DPKNKHSMVNFTHSFYFRGHLCISTELLDMNL
FG10095.1 	IKIIRNKKRFHQQALVEVNILQKLREW0DPKNKHSMVNFTQSFYFRGHLCISTELLDMNL
AN7678.1  	VKIIRNKKRFHQQALIEVNLLQKLKEW~DPHRRHNVVNFTQSFYFRGHLCISTELLGINL
          	:**************:***:****:** **:.:*.:****:***************.:**

MG00760.1 	YEFIKANAFRGFSLKLIRRFTKQMLSSLSLLKQHRVIHCDLKPENILLHHPLHSEIKVID
NCU06638.1	YEFIKSNAFRGFSLKLIRRFTKQMLSSLLLLKQHKVIHCDLKPENILLRHPLHSEIKVID
FG10095.1 	YELIKAHAFRGFSIRIIRRFTKQILSSLNLLKQHKVIHCDLKPENILLRHPLHAEIKVID
AN7678.1  	YEFIKAHDFRGFGIKLIRRFTRQILNTLTLLQAKKVIHCDLKPENILLVHPLSSEIRVID
          	**:**:: ****.:::*****:*:*.:* **: ::************* *** :**:***

MG00760.1 	FGSSCFENEKVYTYIQSRFYRSPEVILGMTYGMPIDMWSLGCILAELYTGVPIFPGENEQ
NCU06638.1	FGSSCFENEKVYTYIQSRFYRSPEVILGMTYGLPIDMWSLGCILAELFTGVPIFPGENEQ
FG10095.1 	FGSSCFENEKVYTYIQSRFYRSPEVILGMTYGMPIDMWSVGCILAELYTGVPIFPGENEQ
AN7678.1  	FGSSCFENEKVYTYIQSRFYRSPEVILGMSYGMPIDMWSLGCILAELYTGYPIFPGENEQ
          	*****************************:**:******:*******:** *********

MG00760.1 	EQLACIMEVFGPPEKHLIEKSTRKKLFFDSMGKPRLTVSSKGRRRRPSSKTLQQVLKCDD
NCU06638.1	EQLACIMEVFGPPEKHLIEKSTRKKLFFDSSGKPRLTVSSKGRRRRPSSKTLQQVLKCDD
FG10095.1 	EQLACIMEVFGPPEKHLIEKSTRKKLFFDSMGKPRLTVSSKGRRRRPSSKTLSQVLKCDD
AN7678.1  	EQLACIMEVFGPPEKHLIEKSTRRKLFFDSLGKPRITVSSKGRRRRPSSKELRQVLKCDD
          	***********************:****** ****:************** * *******

MG00760.1 	EAFLDFLARCLRWDPDRRMKPEEAIRHEFISGIK----ASAPLPRIT-ARDGSPIKRHNT
NCU06638.1	QPFLDFIARCLRWDPDRRMKPEEAVRHEFITGQKTPPSSSMPVPRLASTRESSPSKRVQN
FG10095.1 	EVFLDFLQRCLRWDPERRLKPEDAIRHEFITGRK------MPLPRMPPTRESSPMKRHNT
AN7678.1  	EAFLDFISRCLRWDPQRRLTPSEALRHEFMTGHR-------MAPRPRPFGSQSPGKRANT
          	: ****: *******:**:.*.:*:****::* :         **  .  . ** ** :.

MG00760.1 	IS---TPRPLPEPPGGAGLKTGSN----FARASPQ---------KMITAGSRRASNMSTA
NCU06638.1	LNG--GPRPLPEPPAAAVTTRTSLRSGVVSSGMPQGPLKPTLASGMLPSSRKVSGVTASA
FG10095.1 	IS---TPRPLPDPPGSAVKPLGSLR----SGASPHKP--------VSGPVRKASGQMGPV
AN7678.1  	LSTPTTGRPLPEPPGTSLKNGAVVR-----SRDPSNP---------SPIKATAGKRHSTV
          	:. .:  ****:**. :                *  .                .   ...

MG00760.1 	STVSAATKRVSTGTGTLMMG----------SSNLPRAAGRSASAK----QDLAAAGATAA
NCU06638.1	SGATSGVKRASGGTTGSTAGGFNSGNVGSNGSSLPRAAIRTTSARQQQQQDLAAAGATAA
FG10095.1 	S--SMPTKRTSAGTTGLAQG----------ASGLPRAAGRTVSGK----QDLAAAGATAA
AN7678.1  	SGLPPSTPAKRGINLTTTPG-----------SALPRASARSISGK----PDLATAAAATS
          	*  .  .      .     *           * ****: *: *.:     ***:*.*:::

MG00760.1 	M~N-RRA---------------------------------
NCU06638.1	M~MSRRA---------------------------------
FG10095.1 	M~S-RRS---------------------------------
AN7678.1  	L0LHSRPGTLVDLIRFLRLILTAGSTSQWMRQDTSSYTLT
          	:    *..:  .         ::.:::.   ..::: : :
```
